# Construction and validation of a covariate-based model for district-level estimation of excess deaths due to COVID-19 in India

**DOI:** 10.7189/jogh.14.05013

**Published:** 2024-05-31

**Authors:** Anand Krishnan, Mahasweta Dubey, Rakesh Kumar, Harshal R Salve, Ashish Datt Upadhyay, Vivek Gupta, Sumit Malhotra, Ravneet Kaur, Baridalyne Nongkynrih, Mohan Bairwa

**Affiliations:** 1 Centre for Community Medicine, All India Institute of Medical Sciences, New Delhi; 2 Clinical Research Unit, All India Institute of Medical Sciences, New Delhi; 3 Community Ophthalmology, Dr. Rajendra Prasad Centre for Ophthalmic Sciences, All India Institute of Medical Sciences, New Delhi

## Abstract

**Background:**

Different statistical approaches for estimating excess deaths due to coronavirus disease 2019 (COVID-19) pandemic have led to varying estimates. In this study, we developed and validated a covariate-based model (CBM) with imputation for prediction of district-level excess deaths in India.

**Methods:**

We used data extracted from deaths registered under the Civil Registration System for 2015–19 for 684 of 713 districts in India to estimate expected deaths for 2020 through a negative binomial regression model (NBRM) and to calculate excess observed deaths. Specifically, we used 15 covariates across four domains (state, health system, population, COVID-19) in a zero inflated NBRM to identify covariates significantly (*P* < 0.05) associated with excess deaths estimate in 460 districts. We then validated this CBM in 140 districts by comparing predicted and estimated excess. For 84 districts with missing covariates, we validated the imputation with CBM by comparing estimated with predicted excess deaths. We imputed covariate data to predict excess deaths for 29 districts which did not have data on deaths.

**Results:**

The share of elderly and urban population, the under-five mortality rate, prevalence of diabetes, and bed availability were significantly associated with estimated excess deaths and were used for CBM. The mean of the CBM-predicted excess deaths per district (x̄ = 989, standard deviation (SD) = 1588) was not significantly different from the estimated one (x̄ = 1448, SD = 3062) (*P* = 0.25). The estimated excess deaths (n = 67 540; 95% confidence interval (CI) = 35 431, 99 648) were similar to the predicted excess death (n = 64 570; 95% CI = 54 140, 75 000) by CBM with imputation. The total national estimate of excess deaths for all 713 districts was 794 989 (95% CI = 664 895, 925 082).

**Conclusions:**

A CBM with imputation can be used to predict excess deaths in an appropriate context.

While the number of deaths attributable to the coronavirus disease 2019 (COVID-19) pandemic is a good measure of its impact, the reported country-level COVID-19 death counts have inherent limitations for this purpose due to the weak mortality surveillance systems of many countries and the absence or incompleteness of reporting for indirectly attributable deaths [1]. For this reason, the mortality burden of COVID-19 is best estimated by quantifying the number of deaths during the pandemic period that exceeds the pre-COVID-19 mortality in a given population [2]. This ‘excess death’ methodology has been used to quantify mortality for other pandemics or epidemics, particularly for influenza viruses, both globally and in India specifically [3-5]. There is now a global consensus that the measure of excess death is the most objective indicator of the mortality due to the COVID 19 pandemic [6].

Many global and national estimates of excess all-cause mortality for COVID-19 have been published using different statistical approaches and models [7,8], each with its own strengths and limitations [9]. The Institute of Health Metrics and Evaluation (IHME), using an ensemble of six models, estimated 18.2 million deaths (95% uncertainty interval (UI) = 17.1, 19.6) due to COVID-19 compared to the reported 5.94 million during January 2020 to December 2021 [10]. The World Health Organization (WHO), using a Bayesian Poisson framework, estimated 14.83 (UI = 13.23, 16.58) million excess deaths globally, 2.74 times more deaths than the 5.42 million reported as due to COVID-19 for 2020 and 2021 [11]. These different and non-overlapping estimates raise doubts about their veracity, leading to disputes between researchers, governments, and the general population, as the measure has been interpreted as an indirect indicator of effective management of the pandemic.

Estimating excess deaths is statistically straightforward for populations that have fully functional mortality surveillance systems; the key challenge is doing so in countries with partial or complete lack of data. The WHO report indicated that monthly national data were available for only 100 countries (52%), with other countries having only annual, subnational, or no data, especially among those in Africa and Asia [11]. One of the standard approaches in these situations is to extrapolate using appropriately chosen covariates that are associated with excess death estimates at population level or use data from another country believed to have ‘similar’ characteristics. Both these approaches have limitations and would need validation if the generated estimates were to be credible [12].

In India, the Civil Registration System provides annual district-level data on registered deaths. However, even though death registration is legally mandatory in India, a comparison with the National Family Health Survey data for 2019–21 showed that only 70.8% of deaths were truly registered [13]. According to the data obtained through the medical certification of causes of death in 2020 (which covered 22.5% of all registered deaths), 160 618 deaths were attributable to COVID-19 in India [14]. Consequently, data availability challenges for estimating COVID-19-related mortality in India have been well documented [15].

The WHO estimated that there were 4.74 million excess deaths due to COVID-19 in India, 10 times more than what was officially reported in India (0.48 million) for 2020–21 [11]. Both the IHME and the WHO used subnational data of 12 states for estimation due to issues with data availability, which they then extrapolated to the whole country using covariates [10,11]. However, the Indian government contested the WHO estimate on the grounds of availability and access to mortality data and methods of extrapolation. Undoubtedly, there are wide within-country variations in excess deaths depending on the population profile, health system response attributes, and the pandemic severity, which can raise doubts about the extrapolation [16]. In fact, making estimates for countries without data are a challenge worldwide.

Many studies [17-23] reported population-level covariates for predicting excess deaths in a community. Some of the factors that have been found to be significantly associated include urbanicity; population density; proportion of elderly in the population; presence of specific comorbidities; health system and governance indicators like human resource or bed density; coverage of public health interventions; and indicators of severity of the pandemic. A district-level study of nationally surveyed deaths from India showed that levels of poverty, education, public health access, urban location and other correlates of mortality accounted for one-fifth of differences in age-standardised mortality rates at ages 15–69 years in men and for one-third in women [24]. In a previous study, we found that civil registration data from one district in India was appropriate for excess death estimation [25]. The choice of a district as unit of analysis was due to its size (about 2 million) and availability of most administrative data at this level.

We tested the hypothesis that, in the Indian context, a combination of covariate-based model (CBM) and an imputation approach for the missing covariates can be used to predict excess deaths in districts where mortality data are lacking.

## METHODS

For our analysis, we required data from 2015 to 2019 (minimum of three years) in addition to the data for 2020 and covariate data for all the districts of India. Moreover, while India had 741 districts in 2020 (reference year), we used data from 713 districts as per the year 2015 in this analysis. We combined districts that were split subsequently in the analysis. We extracted data on reported deaths from 2015 to 2020 from the Civil Registration System report for the respective years [26].

We identified a set of covariates based on the literature and likely availability of data at district level. The final list had three state level covariates (state health sector performance score, under-five mortality rate, and population above 60 years), as these data were available only at the state level. The district level covariates covered three domains – the population risk profile (percentage of urban population, density of population per km^2^, prevalence (%) of diabetes, percentage of disadvantaged population-proportion of Schedule caste and schedule tribes, percentage of population living below poverty line); health system capacity or performance (percentage of antenatal care coverage, hospital bed availability per 10 000 population, district hospital score); and pandemic severity (reported number of COVID-19 positive cases and deaths for 2020). We used the district population for 2020 and percentage of deaths registered for 2016–18 as an offset for the analysis. Here we estimated the population for 2020 by multiplying the district level decadal growth rate [27] with the population reported in the 2011 census for each district. We extracted other demographic covariates (percentage of urban population, density of population per km^2^, prevalence (%), and proportion of schedule caste and schedule tribes) and the number of hospital beds per 10 000 population from the District Census Handbook 2011 [28]. Remaining covariates have been collected for 2015–19. Our sources for the covariates were the Report on Healthy States [29], the National Family Health Survey 5 [30], the Census District Handbook [28], the National Multidimensional Poverty Index [31], the Best Practices in Performance of District Hospitals report [32], and COVID-19 India.org [33] (**Table 1**).

**Table 1:** List of covariates identified for district level analyses

Domain	Indicator by domain	Source of information	x̄ (SD)
State level	State health sector performance score	Report on Healthy States [29]	50.1 (12.8)
	Under-five mortality rate per 1000 live births	National Family Health Survey 5 [30]	37.6 (13.4)
	Percentage of population above 60 y	Census District Handbook [28]	8.2 (1.5)
District demographic profile	Population of the district	Estimates for 2020	1 976 521 (1 742 339)
	Percentage of deaths registered in in the year 2016–18	National Family Health Survey 5 [30]	71.6 (20.7)
Population risk profile	Percentage of the district that was urban in 2011	Census District Handbook [28]	26.2 (21.8)
	Percentage of diabetes among adult men	National Family Health Survey 5 [30]	14.4 (5.2)
	Percentage of scheduled caste and tribe population	Census District Handbook [28]	33.0 (23.2)
	Percentage of the population living below the poverty line	National Multidimensional Poverty Index – Baseline Report [31]	25.3 (17.2)
	Density of population per km in 2011	Census District Handbook [28]	696.6 (1795.1)
Health system capacity/performance	Number of beds per 10 000 population	Census District Handbook [28]	104.9 (208.6)
	Percentage of coverage with antenatal care for pregnant women	National Family Health Survey 5 [30]	61.5 (21.1)
	District hospital NITI score	Best practices in performance of district hospitals [32]	11.3 (2.3)
Pandemic severity	Reported COVID-19 deaths in the district by 31 December 2020	Covid19india.org or state government websites [33]	219 (702.7)
	Total COVID-19 cases reported in the district by 31 December 2020	Covid19india.org or state government websites [33]	14 827 (32 001)

x̄ – mean, NITI – National Institution for Transforming India, SD – standard deviation

**Figure 1:**
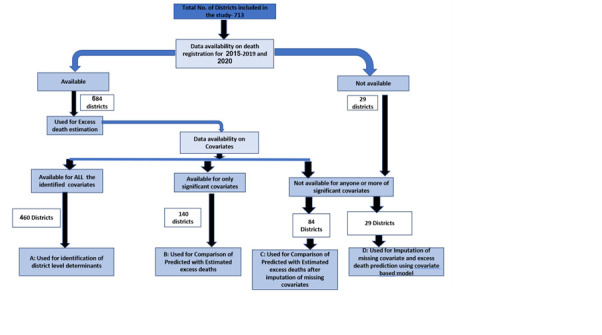
District wise data availability and approach to analysis for estimation of excess deaths adopted in this study.

Based on the availability of data on reported deaths and identified covariates, we divided the districts into four groups, whose characteristics we compared using the χ^2^ test for proportions and the *t* test for mean (**Figure 1**):

- Group A: Minimum three years data available for deaths for 2015–19, death data for 2020 and data on all covariates (n = 460);
- Group B: Minimum three years available for deaths for 2015–19, death data for 2020 and complete data for covariates found significant in the covariate model (n = 140);
- Group C: Data for excess death estimation with incomplete availability of significant covariates (n = 84).
- Group D: Data on deaths not available for 2015–20 and partially (to be imputed) or completely available data for significant covariates (n = 29).

We estimated the expected annual deaths for 2020 for each of the 684 districts (29 did not have data for this analysis) with adequate death data using negative binomial regression model (NBRM) in STATA software, version 17 (StataCorp LLC, College Station, TX, USA), per the following formula:

(f(x;r,*P* = ^x −1^ C*_r_* _− 1_XP*^r^*X(1 − P)x − r))

This is a standard approach used by other researchers, where the expected deaths in a given year is predicted using the margin command after the NBRM for each district [34]. We calculated excess deaths by subtracting the expected deaths for 2020 (as estimated above) from the actual registered deaths. If this value was negative, excess death was taken as ‘0’.

### Association of covariates with excess deaths

This analysis was restricted to the Group A districts (n = 460). We calculated a correlation matrix and except for strong association between the variables related to pandemic severity, (correlation coefficient (β) = 0.91), others had a β of less than 0.70 (Table S1 in the **Online Supplementary Document**). Because of non-normality in the outcome and the likelihood of the zeros coming from more than one source, we opted for a zero inflated negative binomial regression (ZINB) model with district population as an offset [35]. We analysed the association of a dependent variable with the independent variables in four models separately for each domain. We then included the significant variables (*P* < 0.2) for the final model, where any variable with *P* < 0.05 was considered significant. We also considered other models, such as the two-part model (including binary choice and regression models), and zero inflated Poisson regression and regression. We tested the models for fitness per the Akaike information criterion (AIC), which was lowest for the ZINB model (Table S2 in the **Online Supplementary Document**) which was finally used.

### Validation of the covariate-based model

For the Group B districts (n = 140), we predicted the excess deaths by the above CBM and compared its mean and 95% confidence interval (CI) with the previously estimated excess deaths. For Group C Districts (n = 84), we compared the estimated excess deaths with predicted excess deaths after using an imputation method for missing values of significant covariates. If a value of one of the covariates was not available, we imputed its value using a multiple imputation model (20 times imputed data) [36]. For Group D districts (n = 29), we predicted excess deaths based on the validated CBM and imputation method for missing covariates. Thus, we had either a calculated or predicted estimates of excess deaths all districts which were totalled to get the national estimate of excess deaths for COVID-19 for 2020.

## RESULTS

Out of the total 713 districts included in our study, 684 (96%) had a minimum of three-year data apart from 2020 data and were included for the excess death estimation. Twenty-nine districts did not have adequate data on death registration, so the excess deaths due to COVID-19 were predicted rather than estimated (**Figure 1**).

Overall, 460 districts were included in the ZINB model, which covered 15 covariates (**Table 1**). The district-level analysis showed that the deaths registered for 2016–18 accounted for 71.6% of the total deaths; about one-fourths were living below the poverty line; and 8.2% of the population was above 60 years of age. The average district had about two million population, 26% of which was urban, with a density of 696 people per km^2^. There were 104 beds per 10 000 population on average, while the average under-five mortality was 37 per 1000 live births. Furthermore, a district reported 15 000 cases of COVID-19 and 200 deaths on average.

In the final combined ZINB model, five covariates were significantly associated with estimated excess deaths in these 460 districts. Among the state-level covariates, a higher population share of those above 60 years (β = 0.18; 95% CI = 0.06, 0.30, *P* < 0.05) and higher under-five mortality rate (β = 0.016; 95% CI = 0.0030, 0.029, *P* < 0.05) were associated with excess deaths. At the district level, the significant covariates were share of urban population (β = 0.011; 95% CI = 0.004, 0.017, *P* < 0.05), prevalence of diabetes among men (β = 0.02; 95% CI = 0.002, 0.050, *P* < 0.05). and availability of hospital beds (β = 0.0009; 95% CI = 0.0002, 0.001, *P* < 0.05) (**Table 2**).

**Table 2:** Association of state and district level covariates with the number of excess deaths in a district in 2020

	Model 1*		Model 2†		Model 3‡		Model 4§		Combined model‖	
**Independent variable**	**β (95% CI)**	***P*-value**	**β (95% CI)**	***P*-value**	**β (95% CI)**	***P*-value**	**β (95% CI)**	***P*-value**	**β (95% CI)**	***P*-value**
State health sector performance index score	−0.0021 (−0.018, 0.014)	>0.2	Not included in the model						Not included in the model	
Percentage of population above 60 y	0.150 (.0429958, 0.258)	<0.2	Not included in the model						0.18 (0.06, 0.30)	<0.05
Under-five mortality rate	0.016 (−0.0016729, 0.03)	<0.2	Not included in the model						0.016 (0.0030, 0.029)	<0.05
Density of population per km^2^	Not included in the model		−0.00001 (−0.00008, 0.00006)	>0.2	Not included in the model				Not included in the model	
Percentage of share of urban population	Not included in the model		0.009 (0.0030, 0.015)	<0.2	Not included in the model				0.011 (0.004, 0.017)	<0.05
Percentage of Prevalence of diabetes among men	Not included in the model		0.023 (0.0003, 0.045)	<0.2	Not included in the model				0.02 (0.002, 0.05)	<0.05
Percentage of SC/ST population in the district	Not included in the model		−0.006 (−0.013, 0.004)	<0.2	Not included in the model				−0.002 (−0.009675, 0.0053)	>0.05
Percentage of population living below poverty line	Not included in the model		0.01 (0.004, 0.20)	<0.2					0.006 (−0.0052282, 0.0190)	>0.05
Hospital bed availability per 10 000	Not included in the model				0.0011 (0.0003, 0.0019)	<0.2	Not included in the model		0.0009 (0.0002, 0.001)	<0.05
Percentage of coverage with antenatal services	Not included in the model				−0.001 (−0.008, 0.006)	>0.2	Not included in the model		Not included in the model	
District hospital NITI score	Not included in the model				0.04 (−0.03, 0.10)	>0.2	Not included in the model		Not included in the model	
Total COVID-19 cases reported in the district	Not included in the model						5.91 × 10^−6^ (−4.17 × 10^−6^, 0.00001)	>0.2	Not included in the model	
Total number of COVID-19 deaths reported in 2020	Not included in the model						−0.0005 (−0.0010, −3.7 × 10^−6^)	<0.2	−0.00016 (−0.0003, 0.00003)	>0.05

β – correlation coefficient, CI – confidence interval, NITI – National Institution for Transforming India, SC/ST – Schedule Caste/Schedule Tribe

*Model 1 includes the covariates for which data are available at the state level.

†Model 2 includes covariates depicting population risk profile of the districts.

‡Model 3 includes covariates for the health system capacity/performance.

§Model 4 includes covariates for the COVID-19 pandemic severity.

‖Combined model only includes those found significant at 0.2 level in their domain level model with offset for the district population.

**Figure 2:**
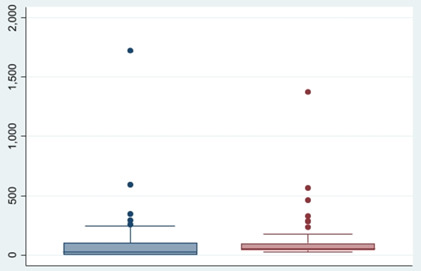
Box plot comparing estimated and predicted mean excess deaths per 100 000 population using covariate model for 140 districts.

We used the above covariate-based model to predict excess deaths in 140 Group B districts. The total estimated excess deaths were estimated at 194 612 (95% CI = 124 294, 264 929) as compared to the CBM-predicted excess deaths of 134 623 (95% CI = 97 996, 171 250), with a large overlap of the 95% CIs. The mean of the predicted excess deaths per district (x̄ = 989, SD = 1588) was not significantly different from the estimated one (x̄ = 1448, SD = 3062) (*P* > 0.05) (**Figure 2**).

**Figure 3:**
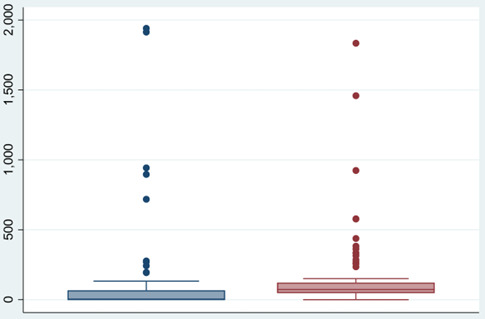
Box plot comparing estimated and predicted mean excess deaths per 100 000 population using Imputation of missing covariates for 84 districts.

We also imputed the missing covariates for 113 districts of group C and D. We used 84 of these districts to validate the imputation model. The estimated excess deaths (n = 67 540; 95% CI = 35 431, 99 648) based on available data were similar to the predicted excess deaths (n = 64 570; 95% CI = 54 140, 75 000) generated by CBM after imputation. The mean of the predicted excess deaths per district (x̄ = 768, SD = 547) for these 84 districts was not significantly different from the estimated one (x̄ = 804, SD = 1675) (*P* > 0.05) (**Figure 3**).

We then kept group A districts as the primary group and compared it with the other three groups. We found that group A districts were larger and had a lower share of northeastern districts, and also noted differences in share of older population, beds per 10 000 population, and share of urban population (**Table 3**).

**Table 3:** Comparison of covariates of districts used for different purposes in the study, presented as x̄ (SD)

	Districts with data used for covariate model						
**Indicator**	**A (n = 460)**	**B (n = 140)**	**C (n = 84)**	**D (n = 29)**	***P*-value**		
Use	Development of CBM	Validation of CBM	Validation of imputed CBM	Prediction using imputed CBM	A vs B	A vs C	A vs D
Percentage of population above 60 years	8.5 (1.4)	7.3 (1.6)	8.3 (1.6)	6.6 (1.4)	<0.05	>0.05	<0.05
Population of the district (in thousands)	2291 (1791)	1 691 (1671)	1 128 (1121)	947 (1384)	<0.05	<0.05	<0.05
Percentage of the district that was urban in 2011	25.1 (18.8)	25.6 (22)	24.5 (19)	52.8 (45.5)	>0.05	>0.05	<0.05
Percentage of diabetes among adult men	14.4 (5.5)	14.7 (4.4)	14.7 (4.5)	15.2 (4.7)	>0.05	>0.05	>0.05
Number of beds per 10 000 population	115.7 (233.8)	74.9 (91.9)	75.8 (193.8)	90.6 (180.1)	<0.05	>0.05	>0.05
Percentage of districts from northeastern India	1.9	48.2	14.2	55.1	<0.05	<0.05	<0.05

CBM – covariate-based model

The estimates place the total excess deaths for the 684 districts for 2020 at 774 282 (95% CI = 644 439, 904 124). The range of excess deaths in districts varied from 0 to 27 117, with 268 (40%) districts not reporting any excess mortality. When expressed per 100 000 population, the estimate varied from 0 to 1940, with a mean of 61.9 (95% CI = 50.12, 73.7) per 100 000 population and a median of 21 (interquartile range = 0, 1049) excess deaths per 100 000 population.

We predicted the excess deaths in the 29 group D districts with imputed missing covariates using the covariate-based model; the total predicted excess deaths were 20 707 (95% CI = 12 865–28 548). Once added to the estimated excess deaths for 684 districts, there was a total of 794 989 (95% CI = 664 895, 925 082) excess deaths nationally for all 713 districts.

## DISCUSSION

In this ecological study, we identified covariates that best explained the district-level excess pandemic death estimates for 2020 using data available in the public domain from nearly all of India’s districts. Among all the selected covariates, urbanicity, prevalence of diabetes mellitus, and hospital bed availability were significantly associated at the district level, and the share of elderly and under-five mortality rate at the state level. The predicted estimate for selected districts using this model was comparable to the estimated excess deaths in those districts. The districts which had problems with data availability on deaths were significantly different on the variables used in the model.

Urban settings have been found to be significantly associated with both reported and excess deaths in various other studies. For example, a study of COVID-19 mortality in the Russian Federation showed greater excess mortality in urban areas [37]. Tamrakar et al. [38] sought to determine district-level correlates of COVID-19 pandemic in India and found significant association of district population density and percent of urban population with COVID-19 infection ratio. This could be because the first wave in India was largely an urban phenomenon and rural areas had been relatively spared.

Meanwhile, being elderly and having a comorbidity such as diabetes were found to be significant individual-level determinants of mortality [39]. It is thus possible that populations with a higher share of the elderly and a higher prevalence of diabetes may also have higher mortality, as confirmed by our analysis. In a state-level analysis of secondary data from India, noncommunicable diseases and their risk factors and epidemiological transition index were associated with reported COVID-19 cases and deaths per million population [40]. In Iran, provinces with a higher level of obesity, cigarette smoking, hypertension, and diabetes mellitus had significantly higher COVID-19 death rates [41]. In a municipal level analysis in Mexico, the prevalence of diabetes and obesity, the human development index, and altitude were associated with higher mortality rate [42].

Intuitively, a higher bed availability in a population denotes better access to inpatient care required for COVID-19, and therefore, a lesser mortality as well. This was seen in a study in the European Union, where the COVID-19 death rate was inversely associated with the number of available general hospitals, physicians, and nurses [43]. However, our analysis showed a positive association with excess deaths. This probably is an artefact created due to cases from neighbouring districts coming to others with a better health service facility. Meanwhile, Anastase et al. [17] found no association between the resuscitation care beds and COVID-19 mortality rate in hospitals during the first and second wave. We also did not detect a significant relation between state performance index, which is not a COVID-19-specific indicator and excess deaths. Cribari-Neto [44] used the differences between the predicted and observed mortality rates as a proxy for local government’s administrative efficiencies, though these were related to COVID-19-specific interventions. In Sweden, the hospitalisations and deaths due to COVID-19 were positively associated with population density and the proportion of immigrants while the proportion of the population aged >65 years showed a negative association [20].

Furthermore, under-five mortality is used as an indicator of socioeconomic development in a developing country, thus reflecting the health care status and quality of life of the population [45]; its significant association with excess deaths in our study is probably an indirect association of health system performance and overall development status of the district.

Environmental factors like air pollution and access to water and sanitation facilities and altitude were also found to be associated with mortality in various studies are environmental factors like air pollution [46,47]. We did not include them in our analysis due to lack of data for those variables and our use of other variables which measures the same domain, such as population risk profile and health system capacity/performance. Importantly, the final list of determinants is dependent on data availability and local context. This does raise questions on using CMB projections for populations from which the equation is not derived. However, our study did show that, despite variations in the distribution of covariates, the prediction by the model was robust and valid.

The national estimate of excess deaths for 2020 in our study was 0.79 million. The ratio this estimate and COVID-19 deaths reported by Government of India (n = 160 618) was 4.8, with the difference being explained by both unreported COVID-19 deaths and deaths indirectly attributed to COVID-19. The ratio between estimated excess mortality rate and reported COVID-19 deaths for India by IHME was 2.95 for deaths between March 2020 to May 2021 [48]. Our estimate is higher than that given by Jha et al. (0.49 million; lower limit: 0.46, upper limit: 0.51) [49], which was based on telephonic household survey of deaths from 1 June 2020 to 31 December 2020. Anand et al. [50] used three methods of estimation and provided the all-cause excess deaths in the range of 1.5 to 3.4 million in wave 1 (from the start of pandemic through March 2021). By comparing the pandemic period to 2019 in 12 Indian states and its extrapolation to the rest of India, Banaji et al. [51] showed a 28% increase in deaths during April 2020 to May 2021 (includes partly the larger second wave). Direct comparisons between these estimates are challenging because of different time periods. Our estimate of 0.79 million excess deaths for 2020 comprises 9.5% of about 8.1 million deaths registered in India in 2020. However, our estimate of 80 excess deaths per 100 000 population, while a substantial increase from the reported, still puts it as much lower than the global average of the cumulative excess death rate as 120 per 100 000 [52].

Our study was based in India, which is the second largest country globally in terms of population and which encompasses widely varying settings, mirroring the diversity often seen at the global level. An average district in India has about two million people, which is larger than 170 countries in the world. Thus, any lessons from this study would have global relevance. The three major estimates available globally are those of the WHO, the IHME, and the Economist, all of which differed significantly from one another. All three analyses used multiple covariates selected from a larger set for basing their estimation in countries, without all-cause mortality data. For such estimations, one’s choice of covariates depends on the scientific plausibility and the coverage of a broad set of domains while keeping their numbers small. While the domains that we have used were identical to the three prior analyses, the actual covariate may vary by context; for example, under the COVID-19-related domains, we included reported COVID-19 deaths and cases as potential covariates, but did not find them to be significant in the final model. This possibly might be because the testing strategy in India was irregular, because there were reporting issues, and because people often moved beyond districts for care or for testing. For any such model, having too many covariates would mean that the related data may also be not available in many countries, which would necessitate imputation, thereby adding one more layer of uncertainty. However, it should be cautioned that these imputations might be based on data which may not hold true during a pandemic. Also, our study clearly demonstrated that districts with better availability of data differ from districts with poor availability, which has implications for extrapolations.

Our study has strengths and limitations. The choice of covariates and the model should be an important consideration. We used a framework with four domains to identify the covariates, though our choice was limited by data availability. We used a ZINB model, as it had the best fit. Other studies have used different models, including beta regression models, while others calculated death rates rather than excess deaths, which need to be adjusted for population size. Our analyses used district-level data from 96% of the districts of India to estimate the excess deaths, with at least three years of pre-COVID-19 data available in public domain to ensure robust estimates. We acknowledge the potential for bias in covariate selection and the implications of using state-level covariates for district-level analysis. The data from the Civil Registration System has its own limitations, including varied coverage over the years and state-level differences in death registrations. Notably, some larger districts attracted patients from neighbouring districts, which was reflected as excess deaths within receiving districts; since we treated less than predicted deaths in sending districts as nil, it is possible that we overestimated the excess deaths in our study. Moreover, the non-availability of some covariate data at the district level forced us to take them at state level (state-level performance, population above 60 years, and under-five mortality rate), which could have affected the analysis of their association.

## CONCLUSIONS

Health system performance, population risk profile, and district health system capacity were among the major determinants of deaths during the pandemic. A model based on select covariates covering these domains can be used to predict excess deaths; in fact, it did well in our study, even if the characteristics of the population differed from that of the original population on which the model was made. However, the covariates and their association with excess deaths are context-specific, and applying these models to populations other than from which they are derived requires caution.

## Additional material


Online Supplementary Document
