# Supplementary material for: Construction and validation of a covariate-based model for district-level estimation of excess deaths due to COVID-19 in India
Source: J Glob Health. 2024 May 31;14:05013. doi: 10.7189/jogh.14.05013 (PMC11140283; doi:10.7189/jogh.14.05013)
Supplement: Online Supplementary Document [file jogh-14-05013-s001.pdf]

Supplementary Tables

Table S1: Correlation matrix of covariates

	ANCcov~H	Preval~s	Hospit~s	Popu~125	density	Totaln~d	Report~o	overal~e	CRScov~t	above6~e	urbanp~s	STST	poor	U5MR_s~e	Scoreo~s
ANCcoverag~H	1.0000														
Prevalence~s	0.3442	1.0000													
HospitalBe~s	0.0846	-0.0636	1.0000												
Populati~125	-0.0694	0.1767	-0.0020	1.0000											
density	0.0276	0.1986	0.0028	0.2126	1.0000										
Totalnumbe~d	0.2050	0.2741	-0.0033	0.5561	0.3335	1.0000									
ReportedCO~o	0.1641	0.1096	0.0220	0.5371	0.3097	0.9119	1.0000								
overallper~e	0.5935	0.2729	0.1752	-0.0508	0.0086	0.2956	0.2325	1.0000							
CRScoverag~t	0.7262	0.2410	0.1431	-0.0646	0.0206	0.2347	0.2097	0.6919	1.0000						
above60yea~e	0.5852	0.4049	0.1064	0.0467	0.0659	0.3138	0.2143	0.5950	0.6187	1.0000					
urbanpopul~s	0.3446	0.2698	0.0390	0.2285	0.3706	0.4498	0.4077	0.3207	0.4141	0.2559	1.0000				
STST	-0.0440	-0.2385	-0.0721	-0.2850	-0.1836	-0.1799	-0.1396	-0.0630	-0.0997	-0.2153	-0.2609	1.0000			
poor	-0.6234	-0.2869	-0.1980	-0.0020	-0.0987	-0.3037	-0.2520	-0.6713	-0.7069	-0.5647	-0.5539	0.2877	1.0000		
U5MR_state	-0.6754	-0.3525	-0.1094	0.1294	-0.0419	-0.2502	-0.1769	-0.8493	-0.6596	-0.5770	-0.3682	0.0520	0.7065	1.0000	
Scoreofcor~s	0.3865	0.2099	0.0737	0.1524	0.0525	0.2360	0.2092	0.3873	0.4745	0.4104	0.2339	-0.1431	-0.4115	-0.3844	1.0000

Table S2: Fitness assessment of different models based on Akaike Information Centre (AIC) values.

Model	AIC
Zero Inflated negative Binomial	5245.72
Two-part model	5737.96
Multiple Linear Regression	8342.63
Zero inflated Poisson	384713.8
